# Match score dataset for team ball sports

**DOI:** 10.1016/j.dib.2024.110625

**Published:** 2024-06-10

**Authors:** Thaksheel Alleck, Tommaso Giovannelli, Luis Nunes Vicente, Roman Mitchell, Ori Remen

**Affiliations:** Department of Industrial and Systems Engineering, Lehigh University, Bethlehem, PA 18015-1582, USA

**Keywords:** Sports analytics, Team ball sports, Match scores

## Abstract

In this data article, we present a dataset containing match scores from major international competitions for 12 popular team ball sports: basketball, cricket, field hockey, futsal, handball, ice hockey, lacrosse, roller hockey, rugby, soccer, volleyball, and water polo. The dataset was obtained by web scraping data available on Wikipedia pages and includes the following information related to individual matches: the year of the competition edition when a match occurred, the names of the two opposing teams, their respective scores, and the name of the winning team. Our match score dataset provides researchers in the field of sports analytics with valuable data that can be used to compute team statistics, develop team ranking and rating systems, infer patterns and trends in a team's performance across the edition years, build predictive models to forecast the outcome of future matches, and evaluate the performance of machine learning algorithms.

Specifications Table

**Table:** 

Subject	Data Science
Specific subject area	Big Data Analytics, Sports Analytics
Type of data	Raw, Filtered
Data collection	Python web-scraping from 161 Wikipedia pages that include match outcomes from different editions of major international competitions selected for 12 team ball sports
Data source location	Lehigh University
Data accessibility	Repository name: Mendeley Data Data identification number: 10.17632/2pt4vmyf27.2 Direct URL to data: https://data.mendeley.com/datasets/2pt4vmyf27/2

## 1 Value of the Data

• The dataset can be used to compute team statistics and develop team ranking and rating systems to evaluate the performance of teams based on their past match scores.
• Statistical and data-driven methodologies can be used to infer patterns and trends in a team's performance across the edition years.
• Researchers in the field of sports analytics can use the dataset to build a predictive model to forecast the outcome of future matches and evaluate the performance of different machine learning algorithms.

## 2 Background

In the last decade, there has been increasing research interest in the analysis of team ball sports [6]. Various avenues have been explored. Some articles use match score data to identify sports with the most random outcomes [1,4]. Others aim to determine scoring patterns across different sports [5]. Finally, there are articles focused on predicting the outcome of sports matches using machine learning algorithms [2,3]. In addition to research papers, several sports datasets have been proposed, and they can be found by simply searching for “Sports datasets” in a web browser. However, such datasets were compiled only for a reduced number of team ball sports, and they are different from our dataset because they cover professional leagues and collegiate tournaments rather than major international competitions. Additionally, such datasets have different formats due to the specificity of each sport, posing challenges for cross-sport analyses.

The information contained in the match score dataset presented in this article covers major international competitions across 12 team ball sports and is highly valuable for researchers in the field of sports analytics. By using and exploring the dataset, researchers can compute team statistics (such as the number of matches won and lost by a team) and develop team ranking and rating systems to evaluate the performance of teams based on their past match scores. By analyzing historical match score data, researchers can gain insights into the strengths and weaknesses of each team across different editions of a competition. Statistical and data-driven methodologies offer the opportunity to infer patterns and trends in a team's performance across the edition years. The dataset can also be used to build predictive models aimed at forecasting the outcome of future matches. By training machine learning algorithms on historical match score data, such models can predict which team is likely to win upcoming matches. Additionally, the dataset enables machine learning practitioners to evaluate the performance of different machine learning algorithms in predicting match outcomes, allowing for the selection of the most effective algorithms for this task.

## 3 Data Description

The dataset we present in this article contains match score data from major international competitions across 12 team ball sports: basketball, cricket, field hockey, futsal, handball, ice hockey^1^, lacrosse, roller hockey, rugby, soccer, volleyball, and water polo. For each sport, the dataset includes the following information related to individual matches: the year of the competition edition when a match occurred, the names of the two opposing teams, their respective scores, and the name of the winning team. Table 1 provides the official names of the competitions selected for each sport, all of which are men's events, and the total number of matches across all the years of the editions of each competition.

**Table 1:** Major international competitions selected for the team ball sports included in our paper, along with the total number of matches across all the edition years of each competition.

Sport	Major international competition	Total number of matches
Basketball	Summer Olympic Games	710
Cricket	ICC Men's Cricket World Cup	474
Field Hockey	Men's FIH Hockey World Cup	645
Futsal	FIFA Futsal World Cup	412
Handball	Summer Olympic Games	412
Ice Hockey	Winter Olympic Games	750
Lacrosse	World Lacrosse Men's World Cup	306
Roller Hockey	World Skate Roller Hockey World Cup	257
Rugby	Rugby World Cup	325
Soccer	FIFA World Cup	852
Volleyball	FIVB Volleyball Men's World Cup	706
Water Polo	FINA Men's Water Polo World Cup	266
	Total:	6115

The complete table with the edition years for each selected competition is provided in Table 4 of Appendix A. Note that each competition occurs periodically, with the frequency varying from every 2 years (such as the World Skate Roller Hockey World Cup) to every 4 years (such as the FIFA World Cup). Some sports have changed their frequency over time, like the FINA Men's Water Polo World Cup, which recently transitioned to a 4-year cycle. Table 5, Table 6, Table 7, Table 8 in Appendix A present the number of matches and teams for each edition. Some popular team ball sports like American football, baseball, and tennis were omitted from our dataset due to either the absence of international competitions or the limited size of their teams compared to the sports included in our paper.

Table 2 introduces some general notation that will allow us to formally describe the match score dataset.

**Table 2:** Notation.

S	Set of sports.
$E_{s}$	Set of editions for the competition selected for sport s∈S.
$P_{e}^{s}=\left\{p_{1},p_{2},\ldots ,\;p_{N_{e}^{s}}\right\}$	Set of all teams playing in edition $e\in E_{s}$.
$N_{e}^{s}$	Cardinality of the set $P_{e}^{s}$.
$M_{e}^{s}\subseteq P_{e}^{s}\times P_{e}^{s}$	Set of matches in edition $e\;\in \;E_{s}$.
$score_{ij}^{s,e}\left(i\right),\;score_{ij}^{s,e}\left(j\right)$	Scores obtained by teams i and j when facing each other, where $\left(i,j\right)\in \;M_{e}^{s}$.
$D_{e}^{s}$	Match score dataset for edition $e\;\in \;E_{s}$, as defined in (3.1).
$D^{s}$	Dataset resulting from the union of the datasets $D_{e}^{s}$ for all $e\;\in \;E_{s}$, as defined in (3.2).

Denoting as S the set of sports, let $E_{s}$ be the set of editions for the competition selected for sport s∈S in Table 1 (one can think of such a set as a set of edition years) and let $P_{e}^{s}=\left\{p_{1},p_{2},\ldots ,\;p_{N_{e}^{s}}\right\}$ be the set of all teams playing in edition $e\;\in \;E_{s}$, where $N_{e}^{s}$ is the total number of teams in that edition. We will denote as $M_{e}^{s}\subseteq P_{e}^{s}\times P_{e}^{s}$ the set of matches in edition e, represented as a set of tuples (i,j), where i and j are opposing teams belonging to $P_{e}^{s}$. For any sport s∈S, edition $e\;\in \;E_{s}$, and match $\left(i,j\right)\in \;M_{e}^{s}$, let $score_{ij}^{s,e}\left(i\right)$ denote the score that team i obtained when playing against team j in edition e (for example, if “5-6” is the outcome of the match (i,j), then $score_{ij}^{s,e}\left(i\right)=5$ and $score_{ij}^{s,e}\left(j\right)=6$) and let $winner_{e}^{s}\left(i,j\right)\in \left\{i,j,Draw\right\}$ represent the winner of the match, where Draw denotes that the match resulted in a tie. We have

$$winner_{e}^{s}\left(i,j\right)=\left\{ \begin{array}{cc} i, & if\;score_{ij}^{s,e}\left(i\right)>\;score_{ij}^{s,e}\left(j\right),\\ j, & if\;score_{ij}^{s,e}\left(i\right)<\;score_{ij}^{s,e}\left(j\right),\\ Draw, & otherwise.\; \end{array}\right.$$

Given a sport s∈S, the match score dataset for each edition $e\;\in \;E_{s}$ can be represented by the following set

(3.1) $$D_{e}^{s}=\left\{\left(e,i,j,score_{ij}^{s,e}\left(i\right),score_{ij}^{s,e}\left(j\right),winner_{e}^{s}\left(i,j\right)\right)\;|\;\left(i,j\right)\in M_{e}^{s}\right\}.$$

Note that given a sport s and an edition e, the size of $D_{e}^{s}$ is equal to the size of $M_{e}^{s}$, which can be obtained from the values in the ‘Matches’ column in Table 5, Table 6, Table 7, Table 8. The size of $P_{e}^{s}$, which is equal to $N_{e}^{s}$, can be obtained from the values in the ‘Teams’ column in Table 5, Table 6, Table 7, Table 8. Combining the match score datasets in (3.1) across all the edition years of the competition selected for each sport s, one obtains the following dataset

(3.2) $$D^{s}=\;\underset{e\in E_{s}}{\cup }D_{e}^{s}.$$

The dataset available in our repository consists of 12 CSV files, each corresponding to a sport s∈S. Such files are named as follows

“match_score_dataset_[SPORT_NAME].csv”,

where [SPORT_NAME] denotes the name of a sport (see the ‘Sport’ column in Table 1). For each sport s, the corresponding CSV file contains the dataset $D^{s}$, as defined in (3.2). Each dataset $D^{s}$ consists of six features, i.e., ‘Edition Year’, ‘Team 1’, ‘Team 2’, ‘Score 1’, ‘Score 2’, and ‘Winner’, which are associated with $e,\;i,j,score_{ij}^{s,e}\left(i\right),score_{ij}^{s,e}\left(j\right),\;$and $winner_{e}^{s}\left(i,j\right)$ in (3.1), where $\left(i,j\right)\in M_{e}^{s}$. To help readers better understand the structure and contents of the dataset at a glance, we include an excerpt from the dataset in Table 3 for the 1974 and 1978 editions of the World Lacrosse Men's World Cup. Note that ‘Team 1’ and ‘Team 2’ represent countries, reflecting our focus on major international competitions.

**Table 3:** Excerpt from the match score dataset for the 1974 and 1978 editions of the World Lacrosse Men's World Cup.

Edition Year	Team 1	Team 2	Score 1	Score 2	Winner
1974	Canada	Australia	18	14	Canada
1974	United States	England	24	10	United States
1974	United States	Canada	26	15	United States
1974	Australia	England	15	3	Australia
1974	United States	Australia	20	14	United States
1974	England	Canada	19	11	England
1978	Canada	England	21	15	Canada
1978	United States	Australia	22	17	United States
1978	United States	Canada	28	4	United States
1978	England	Australia	45	15	England
1978	United States	England	45	71	England
1978	Canada	Australia	16	13	Canada

## 4 Experimental Design, Materials and Methods

For each of the 12 team ball sports included in our paper, to populate the datasets (3.1) and (3.2), we collected real data on match scores from available editions of the major international competitions listed in Table 1. In particular, we obtained match score data through Python web-scraping of 161 Wikipedia pages that include match outcomes from the selected editions of the competitions. Table 9 of Appendix B includes links to the web-scraped pages. Most of the major international sports competitions listed in Table 1 include a group stage, where teams are divided into groups and compete against each other within their group to accumulate points and progress in the competition, and a knockout stage (or bracket stage), where teams are eliminated from the competition if they lose a match. The knockout stage typically consists of the following additional phases: rounds of 16, quarterfinals, semifinals, and finals. The FIVB Men's World Cup for volleyball is an exception to this format. In such a competition, teams are divided into two groups. In the first phase, each team plays one match against all other teams in its group. In the second phase, each team plays one match against all the teams in the other group, and the competition champion is determined based on the total number of points and other criteria.

## Limitations

We note that due to the complexity of the cricket scoring system, scores for cricket matches are not available in the dataset. However, the dataset does include the name of the winning team for each match, as this information is obtainable from Wikipedia pages related to the ICC Men's Cricket World Cup. In 7 matches, the winning team is not available and is listed as “No result”, while in 5 matches, it is labeled “Match abandoned”.

For water polo, the editions of the FINA Men's Water Polo World Cup in 1987, 1989, 1991, and 1997 are missing due to unavailability of the corresponding data.

## Ethics Statement

The authors declare that they have read and followed the ethical requirements for publication in Data in Brief and confirm that the current work does not involve human subjects, animal experiments, or any data collected from social media platforms.

The match score dataset presented in this paper was obtained by web scraping publicly available data from Wikipedia pages. The authors declare that the data collection process adhered to Wikipedia's Terms of Use, which do not contain specific web-scraping policies. The scraped data is non-copyrighted, and there are no privacy concerns associated with it. Links to the scraped pages are included in the paper.

## CRediT authorship contribution statement

**Thaksheel Alleck:** Conceptualization, Methodology, Software, Investigation, Writing – review & editing. **Tommaso Giovannelli:** Software, Validation, Writing – original draft. **Luis Nunes Vicente:** Conceptualization, Writing – review & editing, Supervision, Funding acquisition. **Roman Mitchell:** Validation, Writing – review & editing. **Ori Remen:** Validation, Writing – review & editing.

## Figures and Tables

**Table 4:** Major international competitions and corresponding edition years selected for the team ball sports included in our paper.

Sport	Major International Competition	Edition Years
Basketball	Summer Olympic Games	1964, 1968, 1972, 1976, 1980, 1984, 1988, 1992, 1996, 2000, 2004, 2008, 2012, 2016, 2020
Cricket	ICC Men's Cricket World Cup	1975, 1979, 1983, 1987, 1992, 1996, 1999, 2003, 2007, 2011, 2015, 2019
Field Hockey	Men's FIH Hockey World Cup	1971, 1973, 1975, 1978, 1982, 1986, 1990, 1994, 1998, 2002, 2006, 2010, 2014, 2018, 2023
Futsal	FIFA Futsal World Cup	1989, 1992, 1996, 2000, 2004, 2008, 2012, 2016, 2020
Handball	Summer Olympic Games	1976, 1980, 1984, 1988, 1992, 1996, 2000, 2008,2012, 2016, 2020
Ice Hockey	Winter Olympic Games	1924, 1928, 1932, 1936, 1948, 1952, 1956, 1960, 1964, 1968, 1972, 1976, 1980, 1984, 1988, 1992, 1994, 1998, 2002, 2006, 2010, 2014, 2018, 2022
Lacrosse	World Lacrosse Men's World Cup	1974, 1978, 1982, 1986, 1990, 1994, 1998, 2002, 2006, 2010, 2014
Roller Hockey	World Skate Roller Hockey World Cup	1999, 2001, 2003, 2005, 2007, 2009, 2011, 2013, 2015
Rugby	Rugby World Cup	1987, 1991, 1995, 1999, 2003, 2007, 2011, 2015, 2019
Soccer	FIFA World Cup	1930, 1934, 1938, 1950, 1954, 1958, 1962, 1966, 1970, 1974, 1978, 1982, 1986, 1990, 1994, 1998, 2002, 2006, 2010, 2014
Volleyball	FIVB Volleyball Men's World Cup	1965, 1969, 1977, 1981, 1985, 1989, 1991, 1995, 1999, 2003, 2007, 2011, 2015, 2019
Water Polo	FINA Men's Water Polo World Cup	1979, 1981, 1983, 1985, 1993, 1995, 1999, 2002, 2006, 2010, 2014, 2018

**Table 5:** Tables presenting the number of matches and teams per edition for each competition selected for basketball, cricket, and field hockey.

Basketball			Cricket			Field Hockey		
Edition	Matches	Teams	Edition	Matches	Teams	Edition	Matches	Teams
1964 1968 1972 1976 1980 1984 1988 1992 1996 2000 2004 2008 2012 2016 2020	56 56 57 45 61 47 46 46 46 42 68 38 38 38 26	16 16 17 13 14 13 13 12 12 12 18 12 12 12 12	1975 1979 1983 1987 1992 1996 1999 2003 2007 2011 2015 2019	15 15 27 27 39 37 42 54 48 63 49 58	8 8 8 8 9 12 12 14 16 14 14 10	1971 1973 1975 1978 1982 1986 1990 1994 1998 2002 2006 2010 2014 2018 2023	30 42 42 51 42 42 42 42 42 72 42 38 38 36 44	10 12 12 14 12 12 12 12 12 16 12 12 12 16 16

**Table 6:** Tables presenting the number of matches and teams per edition for each competition selected for futsal, handball, and ice hockey.

Futsal			Handball			Ice Hockey		
Edition	Matches	Teams	Edition	Matches	Teams	Edition	Matches	Teams
1989 1992 1996 2000 2004 2008 2012 2016 2020	40 40 40 40 40 56 52 52 52	16 16 16 16 16 20 24 24 24	1976 1980 1984 1988 1992 1996 2000 2008 2012 2016 2020	32 36 32 36 38 38 44 42 38 38 38	12 12 12 12 12 12 12 12 12 12 12	1924 1928 1932 1936 1948 1952 1956 1960 1964 1968 1972 1976 1980 1984 1988 1992 1994 1998 2002 2006 2010 2014 2018 2022	16 16 12 39 36 36 36 30 28 42 25 27 34 34 34 42 51 32 27 37 26 30 30 30	8 11 4 15 9 9 13 9 8 14 11 12 12 12 13 12 16 14 14 12 12 12 12 12

**Table 7:** Tables presenting the number of matches and teams per edition for each competition selected for lacrosse, roller hockey, and rugby.

Lacrosse			Roller Hockey			Rugby		
Edition	Matches	Teams	Edition	Matches	Teams	Edition	Matches	Teams
1974 1978 1982 1986 1990 1994 1998 2002 2006 2010 2014	6 6 6 6 10 15 28 38 72 56 63	4 4 4 4 5 6 11 18 22 34 40	1999 2001 2003 2005 2007 2009 2011 2013 2015	36 21 24 24 24 24 24 32 48	12 15 16 16 16 16 16 16 16	1987 1991 1995 1999 2003 2007 2011 2015 2019	31 32 31 41 48 8 48 48 38	16 16 16 20 20 8 20 20 17

**Table 8:** Tables presenting the number of matches and teams per edition for each competition selected for soccer, volleyball, and water polo.

Soccer			Volleyball			Water Polo		
Edition	Matches	Teams	Edition	Matches	Teams	Edition	Matches	Teams
1930 1934 1938 1950 1954 1958 1962 1966 1970 1974 1978 1982 1986 1990 1994 1998 2002 2006 2010 2014	18 17 18 22 26 35 32 32 32 38 38 52 52 52 52 64 64 64 64 80	13 16 15 13 16 16 16 16 16 16 17 24 24 24 24 32 32 33 32 32	1965 1969 1977 1981 1985 1989 1991 1995 1999 2003 2007 2011 2015 2019	35 35 42 28 28 28 48 66 66 66 66 66 66 66	11 11 12 8 8 8 12 12 12 12 12 12 12 12	1979 1981 1983 1985 1993 1995 1999 2002 2006 2010 2014 2018	28 28 28 28 12 16 20 20 20 22 22 22	8 8 8 8 8 8 8 8 8 8 8 8

**Table 9:** Links to the web-scraped Wikipedia pages, with [YEAR] indicating the year of the edition of a competition (see the ‘Edition Years' column in Table 4).

Sport	Basketball	Cricket	Field Hockey	Futsal	Handball	Ice Hockey	Lacrosse	Roller Hockey	Rugby	Soccer	Volleyball	Water Polo
[YEAR] Range	1964–1976 1980–2020	1975–2019	1971–2023	1989–2020	1976–2020	1924–2022	1974–2014	1999–2009 2011–2015	1987–2019	1930–2014	1965–2019	1979–2018
Link	https://en.wikipedia.org/wiki/Basketball_at_the_[YEAR]_Summer_Olympicshttps://en.wikipedia.org/wiki/Basketball_at_the_[YEAR]_Summer_Olympics_%E2%80%93_Men%27s_tournament	https://en.wikipedia.org/wiki/[YEAR]_Cricket_World_Cup	https://en.wikipedia.org/wiki/[YEAR]_Men's_Hockey_World_Cup	https://en.wikipedia.org/wiki/[YEAR]_FIFA_Futsal_World_Cup	https://en.wikipedia.org/wiki/Handball_at_the_[YEAR]_Summer_Olympics_%E2%80%93_Men%27s_tournament	https://en.wikipedia.org/wiki/Ice_hockey_at_the_[YEAR]_Winter_Olympics	https://en.wikipedia.org/wiki/[YEAR]_World_Lacrosse_Championship	https://en.wikipedia.org/wiki/[YEAR]_Rink_Hockey_World_Championshiphttps://en.wikipedia.org/wiki/[YEAR]_FIRS_Men%27s_Roller_Hockey_World_Cup	https://en.wikipedia.org/wiki/[YEAR]_Rugby_World_Cup	https://en.wikipedia.org/wiki/[YEAR]_FIFA_World_Cup	https://en.wikipedia.org/wiki/[YEAR]_FIVB_Volleyball_Men%27s_World_Cup	https://en.wikipedia.org/wiki/[YEAR]_FINA_Men%27s_Water_Polo_World_Cup

## Data Availability

Match Score Dataset for Team Ball Sports (Original data) (Mendeley Data).

## APPENDIX

### A International competitions for the considered team ball sports

Table 4 contains the major international competitions and the corresponding edition years selected for the team ball sports included in our paper. Table 5, Table 6, Table 7, Table 8 present the number of matches and teams per edition for each international competition selected for the team ball sports included in our paper.

### B Source of web-scraped data

Table 9 includes links to the Wikipedia pages that were web-scraped to populate our match score dataset.

## Footnotes

1 Despite its use of a puck, we classify ice hockey among team ball sports.
